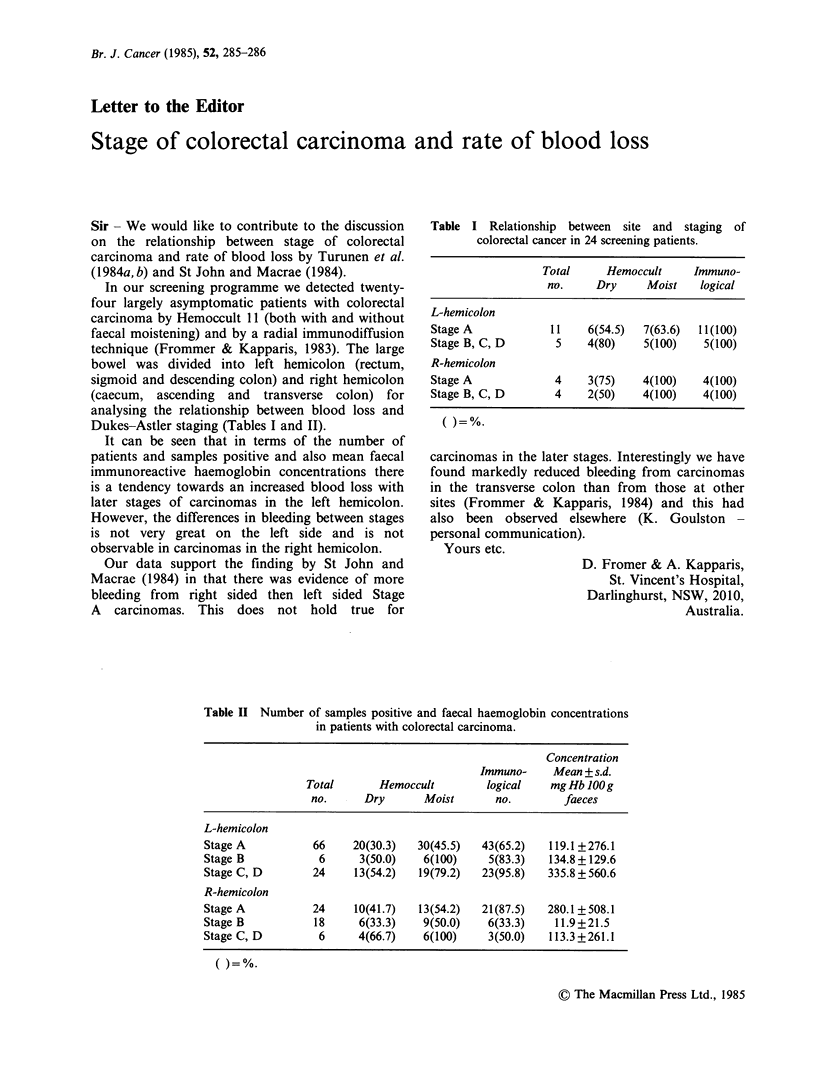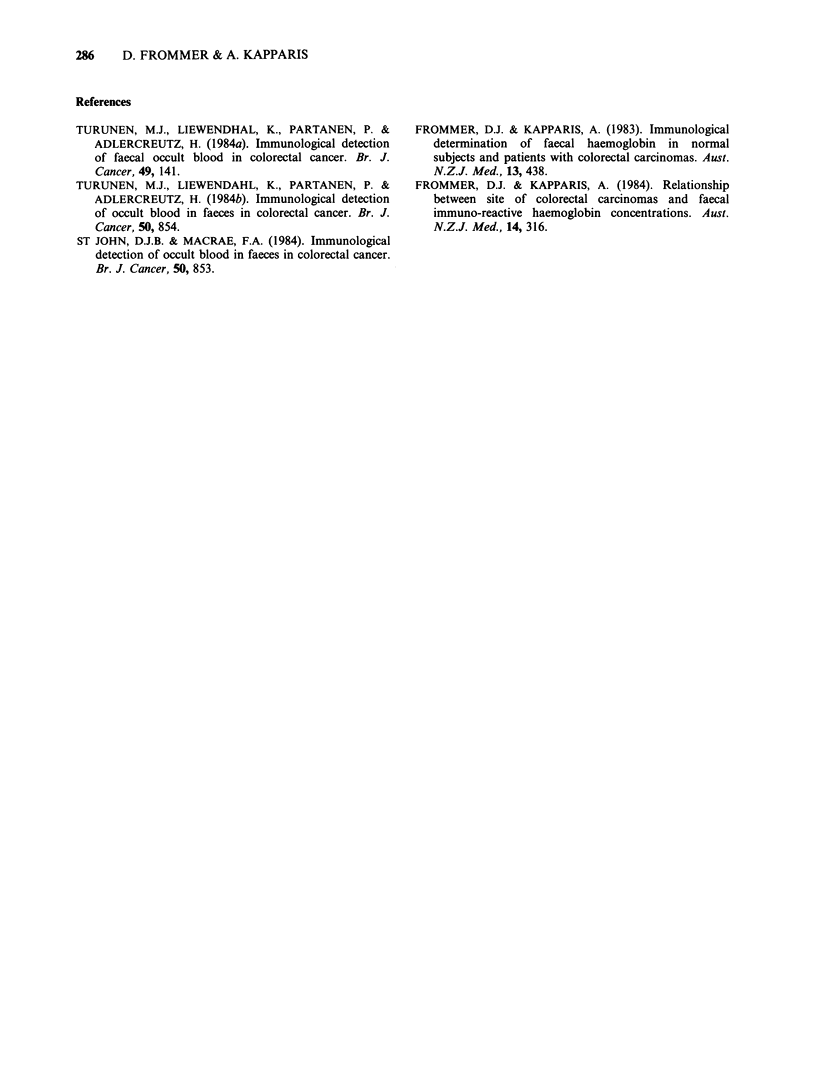# Stage of colorectal carcinoma and rate of blood loss.

**DOI:** 10.1038/bjc.1985.190

**Published:** 1985-08

**Authors:** D. Fromer, A. Kapparis


Br. J. Cancer (1985), 52, 285–286

### Letter to the Editor

### Stage of colorectal carcinoma and rate of blood loss

Sir – We would like to contribute to the discussion on the relationship between stage of colorectal carcinoma and rate of blood loss by Turunen *et al.* (1984*a, b*) and St John and Macrae (1984).

In our screening programme we detected twenty-four largely asymptomatic patients with colorectal carcinoma by Hemoccult 11 (both with and without faecal moistening) and by a radial immunodiffusion technique (Frommer & Kapparis, 1983). The large bowel was divided into left hemicolon (rectum, sigmoid and descending colon) and right hemicolon (caecum, ascending and transverse colon) for analysing the relationship between blood loss and Dukes–Astler staging (Tables I and II).

It can be seen that in terms of the number of patients and samples positive and also mean faecal immunoreactive haemoglobin concentrations there is a tendency towards an increased blood loss with later stages of carcinomas in the left hemicolon. However, the differences in bleeding between stages is not very great on the left side and is not observable in carcinomas in the right hemicolon.

Our data support the finding by St John and Macrae (1984) in that there was evidence of more bleeding from right sided then left sided Stage A carcinomas. This does not hold true for carcinomas in the later stages. Interestingly we have found markedly reduced bleeding from carcinomas in the transverse colon than from those at other sites (Frommer & Kapparis, 1984) and this had also been observed elsewhere (K. Goulston – personal communication).

Yours etc.

D. Fromer & A. Kapparis,
St. Vincent's Hospital,
Darlinghurst, NSW, 2010,
Australia.

**Table I** Relationship between site and staging of colorectal cancer in 24 screening patients.

| | *Total no.* | *Hemoccult Dry* | *Hemoccult Moist* | *Immuno-logical* |
|---|---|---|---|---|
| *L-hemicolon* | | | | |
| Stage A | 11 | 6(54.5) | 7(63.6) | 11(100) |
| Stage B, C, D | 5 | 4(80) | 5(100) | 5(100) |
| *R-hemicolon* | | | | |
| Stage A | 4 | 3(75) | 4(100) | 4(100) |
| Stage B, C, D | 4 | 2(50) | 4(100) | 4(100) |

( ) = %.

**Table II** Number of samples positive and faecal haemoglobin concentrations in patients with colorectal carcinoma.

| | *Total no.* | *Hemoccult Dry* | *Hemoccult Moist* | *Immuno-logical no.* | *Concentration Mean ± s.d. mg Hb 100 g faeces* |
|---|---|---|---|---|---|
| *L-hemicolon* | | | | | |
| Stage A | 66 | 20(30.3) | 30(45.5) | 43(65.2) | 119.1 ± 276.1 |
| Stage B | 6 | 3(50.0) | 6(100) | 5(83.3) | 134.8 ± 129.6 |
| Stage C, D | 24 | 13(54.2) | 19(79.2) | 23(95.8) | 335.8 ± 560.6 |
| *R-hemicolon* | | | | | |
| Stage A | 24 | 10(41.7) | 13(54.2) | 21(87.5) | 280.1 ± 508.1 |
| Stage B | 18 | 6(33.3) | 9(50.0) | 6(33.3) | 11.9 ± 21.5 |
| Stage C, D | 6 | 4(66.7) | 6(100) | 3(50.0) | 113.3 ± 261.1 |

( ) = %.

© The Macmillan Press Ltd., 1985

### References

TURUNEN, M.J., LIEWENDHAL, K., PARTANEN, P. & ADLERCREUTZ, H. (1984*a*). Immunological detection of faecal occult blood in colorectal cancer. *Br. J. Cancer*, **49**, 141.

TURUNEN, M.J., LIEWENDAHL, K., PARTANEN, P. & ADLERCREUTZ, H. (1984*b*). Immunological detection of occult blood in faeces in colorectal cancer. *Br. J. Cancer*, **50**, 854.

ST JOHN, D.J.B. & MACRAE, F.A. (1984). Immunological detection of occult blood in faeces in colorectal cancer. *Br. J. Cancer*, **50**, 853.

FROMMER, D.J. & KAPPARIS, A. (1983). Immunological determination of faecal haemoglobin in normal subjects and patients with colorectal carcinomas. *Aust. N.Z.J. Med.*, **13**, 438.

FROMMER, D.J. & KAPPARIS, A. (1984). Relationship between site of colorectal carcinomas and faecal immuno-reactive haemoglobin concentrations. *Aust. N.Z.J. Med.*, **14**, 316.